# Research paper on abiotic factors and their influence on *Ixodes ricinus* activity—observations over a two-year period at several tick collection sites in Germany

**DOI:** 10.1007/s00436-020-06666-8

**Published:** 2020-03-26

**Authors:** Jörn Gethmann, Bernd Hoffmann, Elisa Kasbohm, Jochen Süss, Birgit Habedank, Franz J. Conraths, Martin Beer, Christine Klaus

**Affiliations:** 1 Friedrich-Loeffler-Institut, Institute of Epidemiology, Südufer 10, D-17493 Greifswald-Insel Riems, Germany; 2 Friedrich-Loeffler-Institut, Institute of Diagnostic Virology, Südufer 10, D-17493 Greifswald-Insel Riems, Germany; 3 Present Address: Institute of Mathematics and Computer Science, University of Greifswald, Walther-Rathenau-Str. 47, 17489 Greifswald, Germany; 4 Friedrich-Loeffler-Institut, Institute of Bacterial Infections and Zoonoses, Naumburger Str. 96a, D-07743 Jena, Germany; 5 Present Address: Brehm Memorial Center, Dorfstraße 22, 07646 Renthendorf, Germany; 6 Umweltbundesamt (German Environment Agency), Health Pests and their Control, Corrensplatz 1, 14195 Berlin, Germany

**Keywords:** *Ixodes ricinus*, Microclimate, Climate change, Tick activity, Temperature, Relative humidity

## Abstract

Tick-borne diseases are a public health issue. To predict vector tick abundance and activity, it is necessary to understand the driving factors for these variables. In this study, the activity of *Ixodes ricinus* was investigated in forest and meadow habitats in Germany with a focus on abiotic factors. *Ixodes ricinus* adults, nymphs and larvae were caught by flagging over a period of 2 years. Microclimatic and weather conditions were recorded at the collection sites. Statistical models were applied to describe correlations between abiotic factors and tick activity in univariable and multivariable analyses. Tick activity was observed in a broad range of air temperature between 3 and 28 °C, and air humidity varied between 35 and 95%. In general, tick activity of nymphs and larvae was higher in forest habitats than that in meadows. With the exception of a single specimen of *Dermacentor reticulatus*, all ticks were *Ixodes ricinus*, most of them nymphs (63.2% in 2009 and 75.2% in 2010). For the latter, a negative binomial mixed-effects model fitted best to the observed parameters. The modelling results showed an activity optimum between 20 and 23 °C for air temperature and between 13 and 15 °C for ground temperature. In univariable analyses, the collection site, month, season, ground and air temperature were significant factors for the number of ticks caught and for all life stages. In the multivariable analysis, temperature, season and habitat turned out to be key drivers. *Ixodes ricinus* positive for RNA of tick-borne encephalitis virus was only found at a single sampling site. The results of this study can be used in risk assessments and to parameterise predictive models.

**Electronic supplementary material:**

The online version of this article (10.1007/s00436-020-06666-8) contains supplementary material, which is available to authorized users.

## Introduction

Tick- and other vector-borne diseases represent a public health issue of increasing importance (Maier et al. 2003; Hartelt et al. 2008; Habedank et al. 2008). Among tick-borne diseases (TBD), Lyme borreliosis (LB) and tick-borne encephalitis (TBE) are of special medical importance. In Europe, both infections are mainly transmitted by the hard tick *Ixodes* (*I*.) *ricinus*. The present climate change (Crowley 2000; Gerstengarbe and Werner 2008; Pachauri et al. 2014) is likely to influence tick activity and the epidemiology of tick-borne diseases in Europe (Dobler et al. 2019; Gray 2008; Süss et al. 2008). Some previous studies documented the spatial (Lindgren et al. 2000; Materna et al. 2008) and temporal distribution of ticks (Dautel et al. 2008); others described a raising incidence of LB and TBE virus (TBEV) and found that the increase was associated with changes in weather and climate (Bormane et al. 2004; Daniel et al. 2008; Lindgren et al. 2006; Süss 2008; Süss et al. 2010; Süss et al. 2008). Further studies suggested that high summer temperatures in Northern Europe might cause an increase in the transmission of tick-borne diseases in late autumn and early spring, as the majority of the tick population becomes active in the latter part of the year (Gray 2008). Empirical long-term studies are required to investigate the possible influence of the climate change on the spatial and temporal distribution of ticks and tick-borne diseases (Eisen 2008). Weather and climate change may also influence the spread of tick-borne diseases such as TBE, as do other factors such as human behaviour or changes in land use that may for example lead to patchy patterns of fields (Randolph 2004; Randolph 2008; Randolph et al. 2008). Korenberg (2009) summarised factors possibly influencing the formation of a natural TBE focus. These factors included virus prevalence, vector occurrence and host activity as well as socio-economic and climate changes.

In this study, we investigated the activity of *I. ricinus* at several sites in forest and meadow habitats in Germany over a period of 2 years. Weather and microclimatic data were recorded at the collection sites to verify the impact of various abiotic factors on tick activity.

## Materials and methods

### Field collection of ticks

**Fig. 1:**
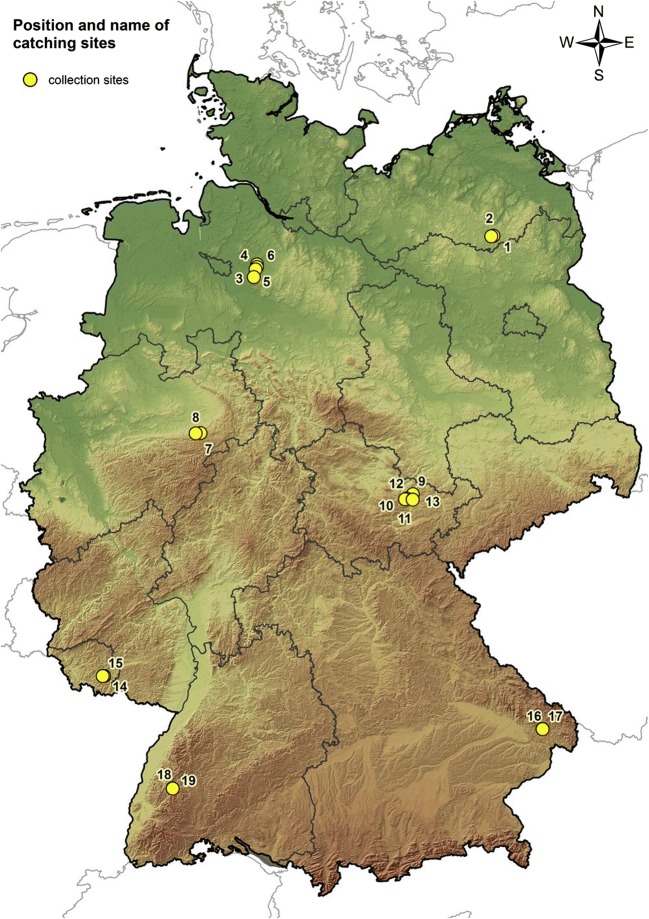
Map of the collection sites in Germany

Ticks were collected in the course of 375 flaggings, which were conducted in a standardised way at 16 sites, located in forest or meadow habitats in seven German federal states in 2009 and 2010 (Fig. 1, Table S1). All sites were situated in TBE risk areas according to the definition of the German Robert Koch-Institut (Robert Koch-Institut 2007, 2019) or in areas where single autochthonous human TBE cases had been reported in the years before.

At each of the 16 sites in Baden-Wuerttemberg (ticks collected only in 2010), Bavaria (ticks collected only in 2010), Saarland, Mecklenburg-Western Pomerania, Lower Saxony and Thuringia, two habitats were studied: one in a deciduous or mixed (but mainly deciduous) forest and another at a wildly grown meadow without grazing animals. In North Rhine-Westphalia, only a habitat in a deciduous forest was examined over the whole time. The selected meadow habitat could only be studied for a short time, because it was intensively used for agricultural purposes, and no other suitable meadow was found to replace it. At each sampling site, four plots were selected, each corner marked with a pole, connected with a marker tape, the plots registered with their coordinates using a global positioning system (Garmin MAP 60CSx, Garmin Deutschland GmbH, Garching, Germany) at a width of 50 m and a length of 50 m (each 2500 square metres) and numbered. In most cases, the plots were bordered directly. Due to the natural conditions at the site, the selection of plots with shared borders was not possible in Thuringia. However, all four plots at a site were located in the same habitat type. Ticks were collected in the plots by flagging with a blanket of about 1 × 1 m^2^ in a meandering way over a defined and documented time (mostly 30 min) by well-trained persons (mostly by two people at each site). During each flagging event, two plots were examined per habitat and by one person each. Tick activity was described as collected ticks per person and hour. Ticks were identified as adult female or adult male ticks, nymphs or larvae by morphological criteria. Species determination was conducted for all ticks according to Estrada-Peña et al. (2004) and the specimens stored at − 80 °C for further investigations.

Except for the sites in Thuringia, all sites were flagged every 6 weeks during the whole year. Flagging was not performed at temperatures below 0 °C, when there was snow or if it was too wet. In Thuringia, the plots were flagged nearly every 2 to 4 weeks. When the larvae were equally distributed on the flag, the total number was extrapolated based on the counted number of larvae in one quadrant.

### Recorded data

At each flagging event, the following parameters were recorded: ground temperature and ground humidity (‘dry’, ‘moist’, ‘wet’), air temperature and air humidity (measured 30 cm above ground), cloud coverage (‘sunny’, ‘sunny-cloudy’, ‘cloudy’), and wind speed (‘windstill’, ‘slightly windy’, ‘windy’) (see Table 1 for all parameters).

**Table 1:** Definition of the parameters for the tick catching

Definition of the parameters			
Parameter	Method	Possible values	Definition
Ground humidity	Measured by putting an absorbent paper on the ground	Dry	No water on paper
		Moist	Water on paper after 5–10 min
		Wet	Water on paper after 0–5 min
Air temperature	Measured 30 cm above ground with ‘Luftfeuchte-und Temperaturmessgerät TROTEC, Trotec GmbH, Heinsberg, Germany’	Continuous (numeric)	
Ground temperature	Measured with Allzweck thermometer, Carl Roth GmbH and Co KG, Karlsruhe, Germany	Continuous (numeric)	
Air humidity	Measured 30 cm above ground with ‘Luftfeuchte- und Temperaturmessgerät TROTEC, Trotec GmbH, Heinsberg, Germany’	Continuous (numeric)	
Wind speed	By observing the effect of wind on the flag	Still	No flag movement by wind
		Slightly windy	Flag slightly moved by wind
		Windy	Flag heavily moved by wind
Cloud coverage	By observing the sky	Sunny	No clouds
		Sunny-cloudy	Some clouds, but sun still visible
		Cloudy	Sky fully covered by clouds
Habitat	Determined by plants growing at sampling site	Forest	Habitat is dominated by trees
		Meadow	Habitat is dominated by grassland
Date	Use of calendar	Days	
Daytime	Determined by time of flagging	Morning	6–10 a.m.
		Noon	10 a.m.–2 p.m.
		Afternoon	2 p.m.–6 p.m.
Duration	Duration of flagging in minutes	Numeric	
Number of collecting persons	Number of persons involved	Discrete	
Collection site	Determined by expert opinion		
Federal state	Federal state, where the collecting site was located	Discrete (16 federal states)	

Air temperature and humidity were measured (Luftfeuchte- und Temperaturmessgerät TROTEC, Trotec GmbH, Heinsberg, Germany) as well as ground temperature (Allzweck thermometer, Carl Roth GmbH and Co KG, Karlsruhe, Germany).

In addition to these abiotic factors, the type of habitat (forest or meadow), date, daytime (‘morning’, ‘noon’, ‘afternoon’), duration, number of collecting persons, designation and the geographic coordinates of the collection site were recorded.

### Detection of TBEV-RNA in ticks

As the TBEV prevalence may be a marker for increased tick activity and the distribution of ticks in space and time, the collected ticks were checked for TBEV-RNA. All collected ticks were examined by two different real-time RT-PCRs for TBEV-RNA: a PCR according to Schwaiger and Cassinotti (2003) was used in a modified version (Klaus et al. 2010b) and confirmed by an independent TBEV assay (Klaus et al. 2010a). For PCR, the ticks were individually ground in a mixer mill (Retsch, Haan, Germany) with three stainless steel beads and 400 μl medium (MEM Earle, Biochrom AG, Berlin, Germany). Aliquots of the suspensions were pooled (50 μl each from 10 adults/nymphs or 20 larvae) and RNA extracted using the NucleoSpin® 96 Virus kit (Macherey-Nagel, Düren, Germany) according to the manufacturer’s instructions on an automated liquid handling station (Star, Hamilton Star, Bonaduz, Switzerland). All RNA samples were stored at − 80 °C to avoid RNA degradation. If a pool was TBEV-positive, RNA was extracted from the individual ticks and checked for the presence of TBEV-RNA. Previous work had demonstrated that it was possible to detect a single TBEV-positive tick in a pool (Klaus et al. 2012).

### Statistical analysis

In a first step, we carried out explorative and descriptive statistical analyses to assess the influence of single factors on the number of collected ticks. Potential associations and correlations were tested using a correlation analysis. The factors were tested for statistical significance in a univariable approach by means of Kruskal-Wallis tests.

The optimal temperature for tick activity (tick activity optimum) was estimated by means of a second-order polynomial model. The temperature optimum represents the peak of the polynomial model. To calculate it, we set the first derivate to 0 and solved it to x.

With respect to time data, only month and year of collection were used for statistical analysis. In addition, the season was derived from the month of collection.

In the next step, negative binomial mixed-effects models (R package glmmTMB (Brooks et al. 2017)) were fit to the data to examine the combined influence of habitat and weather parameters on the number of collected ticks in a multivariable analysis. The subpopulations of larvae, nymphs, adult female and adult male ticks were modelled separately. Insights from the first step were used to specify the regression models as described below. Poisson regression and negative binomial regression models are often used to analyse count data. Poisson regression assumes that the mean and variance of the response variable are equal, whilst this assumption is less strict in negative binomial regression, which can be regarded as a generalisation of the Poisson regression. For each of the four subpopulations, the variance of the data was considerably greater than the mean (i.e. overdispersion; e.g. residual deviance in female adult ticks was 2321.0 with 364 degrees of freedom, test for overdispersion was significant with *p* value = 0.001364) (Cameron and Trivedi 1990; Cameron and Trivedi 2005; Cameron and Trivedi 2013). Therefore, we chose the negative binomial regression for further modelling.

Furthermore, because of the high proportion of zero counts in the data (larvae, 68%; nymphs, 9%; female adult ticks, 41%; male adult ticks, 34%), we compared our approach to zero-inflated regression models.

For the negative binomial regression model, we included all abiotic factors that had been significant in the Kruskal-Wallis tests in the first step, i.e. (i) air temperature (quadratic term), (ii) ground temperature (quadratic term), (iii) ground humidity for at least one of the four subpopulations, (iv) type of habitat and (v) season (Fig. S1). For the zero-inflated models, the probability for excess zeros was assumed to be constant. To take a potential influence of the flagging sites and repeated measurements at the same site into account, we included random effects for the sites in the models. Moreover, we used an offset for the intensity of sampling (intensity = number of sampling persons × duration per person in minutes / 60), because the number of persons who had performed the flagging and the sampling time had not always been identical.

Competing models were compared using the Akaike information criterion (AIC) and likelihood ratio tests.

For all statistical analyses, we used the R statistical software (RCore 2015) with the packages ggplot2 (Wickham 2016), glmmTMB (Brooks et al. 2017), MASS (Venables and Ripley 2013), lme4 (Bates et al. 2015), pscl (Zeileis et al. 2008) and lmtest (Zeileis and Hothorn 2002).

## Results

### Descriptive statistics and univariable tests

**Fig. 2:**
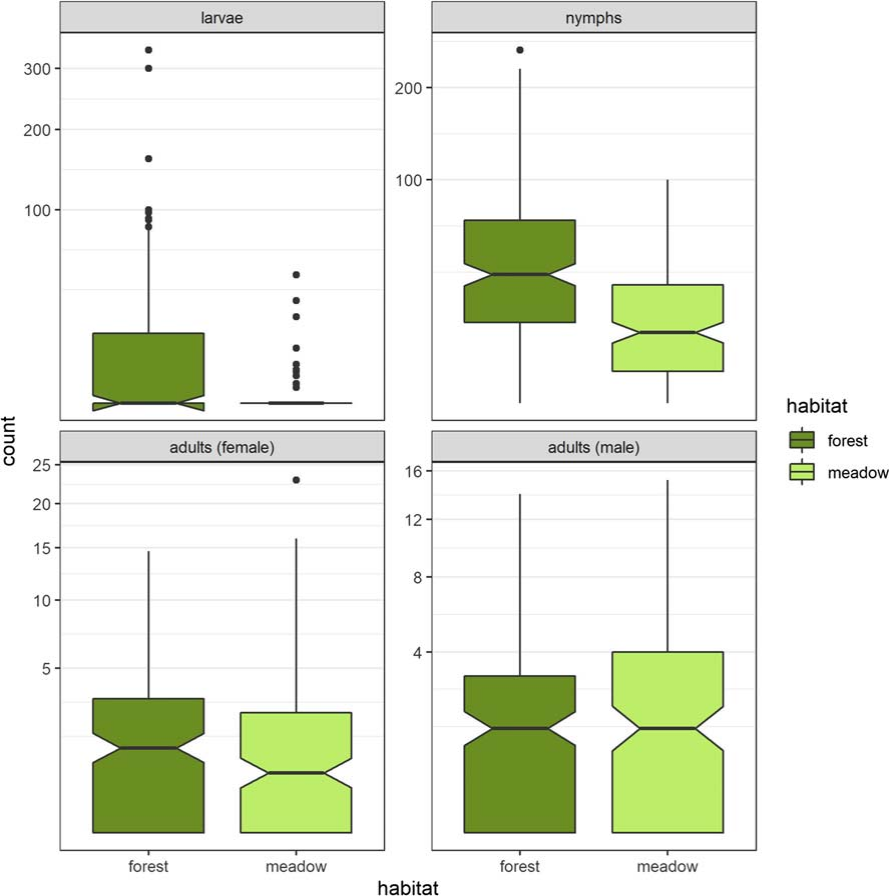
Numbers of ticks collected per habitat and age group (per person and hour)

In 2009 and 2010, a total of 17,630 ticks were collected at 375 flagging events at all sites (Table S2). All ticks but one were identified as *I. ricinus*. The remaining tick was a female *Dermacentor reticulatus*; it was found in Thuringia. Besides, in several flagging events, some subpopulations were not found (females in 41.1%, males in 33.6%, nymphs in 9.1% and larvae in 68.3%). Often, only a few adult ticks were caught (see Fig. 2). Most of the collected ticks were nymphs (63.2% in 2009 and 75.2% in 2010).

**Fig. 3:**
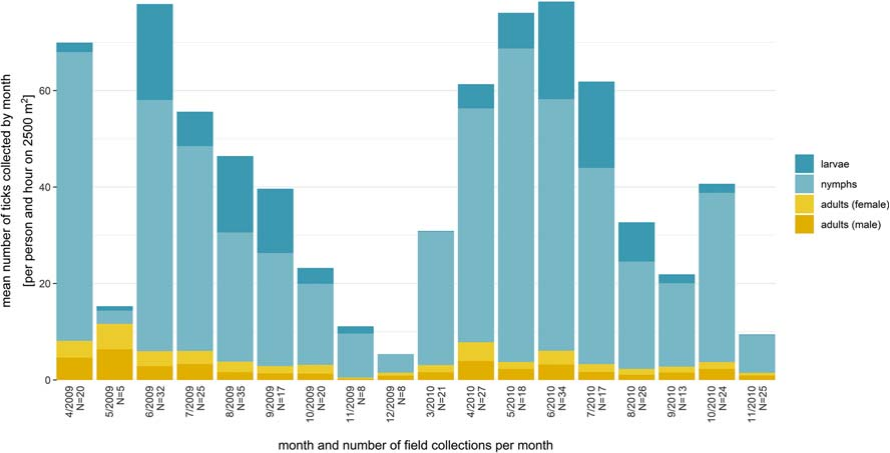
Total numbers of ticks collected by month and age group

**Fig. 4:**
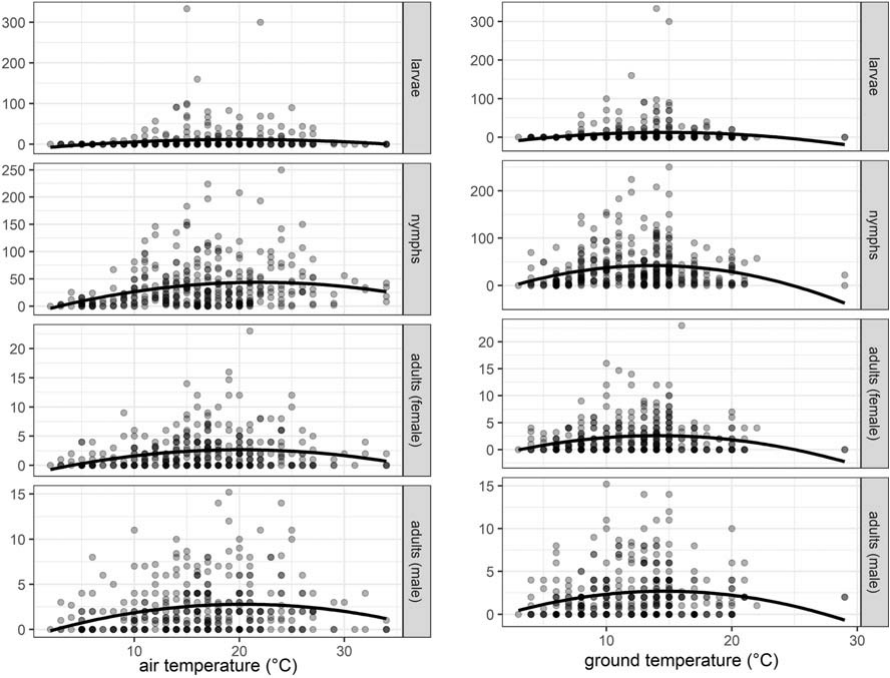
Temperature and numbers of collected ticks (per person and hour). The blue line shows the local regression fit (loess regression)

Tick activity was observed in a seasonal pattern at an air temperature between 3 and 28 °C and at a ground temperature between 4 and 22 °C (Figs. 3 and 4). It could be detected at very low temperatures (3 °C air temperature and 4 °C ground temperature), but at a low level (up to six adult ticks or nymphs, up to 38 larvae). A second-order polynomial model describing the temperature dependency revealed an activity optimum between 19 and 23 °C for air temperatures and a ground temperature between 13 and 15 °C (Table 2, Fig. 4). Information on relative air humidity was available for 224 out of the 375 datasets. Tick activity was observed between 35 and 95% of relative humidity (Fig. S2).

**Table 2:** Temperature optimum for tick activity based on a second-order polynomial model (by calculating the temperature inflection point for each group)

Tick stage and gender	Air temperature	Ground temperature
Larvae	20.11	14.68
Nymphs	21.97	13.72
Female adults	20.16	14.06
Male adults	20.43	14.68

Univariable tests were performed for the parameters federal state, site, habitat, year, month, season, daytime, air temperature, ground temperature, humidity, cloud coverage and wind. Statistically significant differences were observed for the variables federal state (larvae and nymphs only), site, habitat (larvae and nymphs only), year (larvae only), month, season, air temperature, ground temperature and humidity (nymphs and female ticks only). The results are shown in Table 3. Tick activity differed statistically significant between the two habitats ‘forest’ and ‘meadow’ (Fig. 2, Table 3) for nymphs and larvae, but not for adult ticks. The mean numbers of adult ticks collected per month and normalised by person and hour was highest in the habitat ‘meadow’ in Thuringia (36.5, 72.7, 34.9 and 30.3 adult ticks per person and hour in April, May, June and July). In comparison, the mean number of adult ticks collected at all other sites was 8.4, 4.0, 7.5 and 5.5 ticks per person and hour in each month between April and July, with a maximum of 31.6 in June in the habitat ‘forest’ in Baden-Wuerttemberg.

**Table 3:** Tests for significant differences in categorical variables (Kruskal-Wallis test with *p* value correction)

	Larvae				Nymphs				Adult female ticks				Adult male ticks			
Variable	Statistic	df	*p* value	*p* (Bonf.)	Statistic	df	*p* value	*p* (Bonf.)	Statistic	df	*p* value	*p* (Bonf.)	Statistic	df	*p* value	*p* (Bonf.)
Federal state	39.83	6	< 0.001	< 0.001	103.21	6	< 0.001	< 0.001	19.79	6	0.003	0.13	18.76	6	0.005	0.20
Site	78.53	15	< 0.001	< 0.001	164.22	15	< 0.001	< 0.001	60.02	15	< 0.001	< 0.001	45.31	15	< 0.001	0.00
Habitat	50.57	1	< 0.001	< 0.001	58.79	1	< 0.001	< 0.001	5.41	1	0.02	0.879	1.363.00	1	0.243	1.00
Year	17.76	1	< 0.001	0.001	1.53	1	0.216	1.00	2.95	1	0.086	1.00	0.042	1	0.838	1.00
Month	41.56	9	< 0.001	< 0.001	70.04	9	< 0.001	< 0.001	51.13	9	< 0.001	< 0.001	55.49	9	< 0.001	< 0.001
Season	26.49	3	< 0.001	< 0.001	35.88	3	< 0.001	< 0.001	36.61	3	< 0.001	< 0.001	28.11	3	< 0.001	< 0.001
Daytime	0.05	2	0.976	1.00	3.05	2	0.218	1.00	0.211	2	0.9	1.00	1.06	2	0.59	1.00
Air temperature (categorial)	17.70	2	< 0.001	0.006	45.78	2	< 0.001	< 0.001	20.45	2	< 0.001	0.002	17.11	2	< 0.001	0.01
Ground temperature (categorial)	15.19	2	< 0.001	0.022	31.97	2	< 0.001	< 0.001	21.70	2	< 0.001	< 0.001	27.52	2	< 0.001	< 0.001
Ground humidity	6.23	2	0.044	1.00	22.24	2	< 0.001	< 0.001	18.84	2	< 0.001	0.00	6.10	2	0.047	1.00
Cloud coverage	6.36	2	0.042	1.00	1.94	2	0.378	1.00	2.17	2	0.338	1.00	0.247	2	0.884	1.00
Wind	3.91	2	0.141	1.00	1.42	2	0.491	1.00	0.113	2	0.945	1.00	2.90	2	0.234	1.00

*Statistic* Kruskal-Wallis rank sum statistic, *df* degree of freedom, *p value p* value of the test, *p* (*Bonf*.) the Bonferoni corrected *p* value of the test

No statistically significant differences in tick activity were found for the time of the day (morning, midday and afternoon), wind and the years 2009 and 2010 (Table 3). Only the numbers of collected and estimated larvae differed between 2009 and 2010.

#### Regression models

All parameters with a statistically significant result in at least one group in univariable analysis were included in the multivariable model, i.e. season (winter, spring, summer and autumn), habitat (forest, meadow), air temperature, ground temperature and ground humidity (dry, humid, wet) as fixed effects. The parameter ‘site’ was added as a random variable. Since the variables ‘federal state’ and ‘site’ were related, only ‘site’ was included. Furthermore, we excluded the variable ‘month’ from the model, as it is highly correlated with the variable ‘season’. We also excluded the variable ‘year’ since it differed significantly only for those larvae where the number of individuals had partially been estimated. The results are shown in Table 4.

**Table 4:**
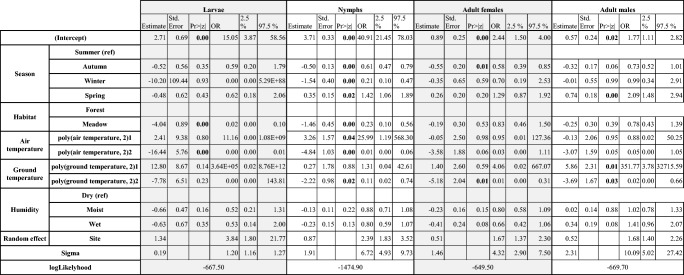
Results of the negative binomial model for adult females, adult males and nymphs

*Estimate* the estimates of the model, *Std. Error* standard error, *Pr > |z| p* value, *OR* odds ratio, *2.5%* lower confidence interval, *97.5%* upper confidence interval)

For female adult ticks, significant effects on their number were found for ground temperature (quadratic term) and season (in autumn, the numbers of collected ticks were reduced in comparison to summer).

For male adult ticks, significant effects on their number were found for the parameters ‘ground temperature’ (linear and quadratic term) and season (in spring, the numbers of collected ticks were increased as compared to summer).

With respect to nymphs, statistically significant effects were found for all parameters (season, habitat, air temperature and ground temperature) as all variables showed significant effects in the negative binomial model component. In detail, in autumn and winter, a statistically significant decrease in the number of nymphs was found, whereas during spring, there was an increase in the number of nymphs in comparison with summer, and meadows were associated with a decreased number of collected nymphs in comparison with forests.

### Testing of ticks for TBEV-RNA

All *I. ricinus* ticks were checked in pools for TBEV-RNA. Only in Bavaria, a single positive pool of female ticks was detected, collected at the site Loderhof in the forest habitat. Seven out of ten ticks that belonged to this pool tested positive for TBEV-RNA.

## Discussion

Biotic and abiotic factors have substantial impact on the life cycle of hard ticks. Among the biotic factors, the available hosts represent a key factor, which is essential, as the ticks require blood before they can moult and reach the next stage in their life cycle. A broad spectrum of suitable host species and an abundant number of various species of wild life are present at the study sites. Thus, they do not represent a limiting factor for tick development. Abiotic factors such as temperature and humidity, however, may restrict the development of hard ticks, in particular *I. ricinus* (Dautel 2010; Dautel et al. 2016; Kiewra et al. 2014). Süss et al. (2008) described questing ticks at temperatures as low as 7 °C. Dautel (2010) found that torpor of ticks took place between 0 and 10 °C depending on the tick species. For *I. ricinus*, it has been assumed that the lower temperature limit may be relevant as tick activity was observed near Berlin during the whole winter 2006/2007 in Germany (Dautel et al. 2008). Earlier experimental studies showed that the microclimate can influence both tick development and TBEV prevalence (Danielová et al. 1983). In this study, it became evident that higher temperature (24 °C) and increased relative air humidity (97%) influenced tick development positively compared with lower temperatures (15 °C) and lower relative air humidity (75%). Andreassen et al. (2012) described for seven sites in Norway that relative humidity and temperature had an impact on the TBEV prevalence in ticks. Increasing tick activity was observed for three sites in the Siebengebirge, Germany, between 1978 and 2008, most likely supported by the local climatic conditions (Schwarz et al. 2012). Burtis et al. (2016) observed that the number of *I. scapularis* collected by flagging decreased during hot and dry weather, but the prevalence of ticks infesting small mammals was not influenced. A certain range in temperature and humidity may therefore be beneficial for the development of ticks, especially larvae.

In our study, tick activity was observed in a broad range of temperatures varying from 3 to 28 °C and air humidity between 35 and 95%. However, in our study, a low temperature of 3 °C or a low humidity of 35% was sufficient for some ticks to quest for hosts. Schulz et al. (2014) detected single questing *I. ricinus* even at 1.1 °C ground temperature. Süss et al. 2008 determined the optimum for tick activity to be at 85% relative humidity. However, Schwarz et al. (2012) observed tick activity even at approximately 45% relative humidity. Gilbert et al. (2014) showed that *I. ricinus* ticks can be resilient to climate change. For example, more ticks from cooler climates (Scotland) quested at lower temperatures than ticks from warmer regions (France). So, the critical temperature for *I. ricinus* of 32 °C (Eisen et al. 2016) may increase in the future by adaptation of the ticks.

We found that nymphs were more abundant in forest habitats. At most of the sites, their numbers were more than two-fold higher than that on the corresponding meadows. *Ixodes ricinus* is known to be widespread in deciduous woodlands in Europe. Here, this tick occurs in higher abundances, for example, in forests consisting of a mixture of beech and oak trees with shrubbery, a well-developed herb layer and leaf litter, where the ticks can find shelter and where hiding and resting places exist for a large variety of small and large vertebrate hosts. The shadow and the canopy of the trees at the forest sites represent a more favourable microclimate than the meadows. So, ticks may be able to avoid dehydration in the forest habitat more successfully. Only at the forest site in Thuringia, this was not evident, as tick activity was very low there during the whole study period, although the habitat was similar to other forest habitats. We have no clear explanation for this result, but it seems possible that other factors such as a low number of small mammals limited tick development at the sites in Thuringia. However, this was not investigated in this study.

Tick activity decreased markedly in late autumn and winter, in particular at the forest sites, but also on meadows. At the latter sites, the numbers of questing ticks were also low in summer, presumably due to high temperatures and low humidity in these habitats. In Northern Europe, two peaks of activity were found, i.e. in early summer and early autumn (Cayol et al. 2017). However, this phenomenon could not be confirmed in our study. In Hungary, a major peak in spring was recorded (Egyed et al. 2012), but the differences in tick activity between six habitats were small.

The design of this study did not allow the detection of differences in tick activity during the day as the variation of data was too large. To overcome this difficulty, ticks should be collected more frequently during the day (e.g. every 1 or 2 h) at selected sites in future studies.

Nearly three out of four collected ticks in this study were nymphs (75%). However, in most previous studies, only nymphs and adult ticks had been collected. Therefore, the observed proportion between the different age groups may not be fully comparable to those we found. Kazimírová et al. (2016) for example found 83.1% of the collected ticks to be nymphs. Mehlhorn et al. (2016) collected 4013 *I. ricinus* (nymphs and adults) in two German federal states within 2 years, 66.4 to 87.3% being nymphs.

In recent studies, nymph density was investigated as a potential indicator for the risk of tick-borne diseases and for producing high-resolution risk maps for Baden-Wuerttemberg (Boehnke et al. 2015) and Germany (Brugger et al. 2016). These investigations emphasise that nymphs may be the most important tick stage for the transmission of pathogens.

It should be noted that the numbers of larvae was extrapolated in some cases (*n* = 14). As the number of the estimations is low compared with the total number of flagging events (*n* = 375), the influence on the model outcome is most probably small. Nevertheless, the results for larvae should be interpreted carefully.

Only one tick pool was positive for TBEV-RNA, although clinical cases of TBEV occurred in the surroundings of the collection sites. Although it is not difficult to find questing ticks in their habitats, testing ticks for TBEV with its natural microfoci is not sufficient to assess the risk of TBE transmission in a region. Recent studies highlighted that the chance to detect TBEV in natural foci may be very small (Kupča et al. 2010). To detect potential natural foci of TBEV, it has been recommended to test at first animals as sentinels by antibody detection assays followed by targeted tick collection, if positive sera have been found (Frimmel et al. 2019; Klaus et al. 2013). Testing rodents as the most important hosts of TBEV may also help to detect natural foci (Achazi et al. 2011).

The combined influence of selected abiotic factors on tick activity was modelled using negative binomial regression models. This regression type was chosen with respect to the characteristics of the data, i.e. the presence of overdispersion and the high number of zero counts. Other choices could have included Poisson regression and zero-inflated negative binomial regression. However, the lowest AIC was achieved by using negative binomial regression. Only for the counts on larvae, where the highest proportion of zero counts occurred, a zero-inflated regression model achieved a slightly lower AIC than the respective negative binomial regression model, for example, using a zero-inflation term depending on ground temperature (linearly and quadratically, AIC 1348.5).

Nevertheless, due to the limited numbers of observations, we preferred the less complex model to avoid potential overfitting.

Although the methods we used for measuring tick activity are well-established, we strongly recommend that ticks are only collected by trained and experienced personnel and that standardised procedures are used to obtain comparable results.

Further investigations are needed to improve our understanding of the influence of the life stages of ticks, their development and their competence as vectors, including long-term studies in selected habitats, which include biotic (e.g. host density) and abiotic factors (in particular temperature and humidity) in addition to experimental infections of ticks and hosts with tick-borne pathogens in the laboratory.

In our study, ticks were found to be active in a wide temperature range, and there were several key factors for tick activity, e.g. temperature or habitat. Whilst air temperature has a major impact on nymphs and larvae, the ground temperature is important for adult ticks. A temperature optimum was found at about 19–23 °C for air temperature and about 13–15 °C for ground temperature. These findings are important for risk assessments and may for example help to estimate the risk for being bitten by ticks, and can thus be used in predictive models.

We also observed substantial variability in tick activity between different catching sites. Possible reasons might be differences in the microhabitats (e.g. soil types, plant species) and in the available hosts. Further analyses are required to understand the activity of ticks in more detail.

## Electronic supplementary material

Figure S1 Numbers of ticks caught per humidity and age group [per person and hour] (JPEG 884 kb)

Table S1 Sites for measuring tick activity (DOCX 13 kb)

Table S2 Ticks collected in 2009 and 2010 at all sites (DOCX 14 kb)
