# Supplementary figures and images for: Research paper on abiotic factors and their influence on Ixodes ricinus activity—observations over a two-year period at several tick collection sites in Germany
Source: Parasitol Res. 2020 Mar 26;119(5):1455–66. doi: 10.1007/s00436-020-06666-8 (PMC7184057; doi:10.1007/s00436-020-06666-8)

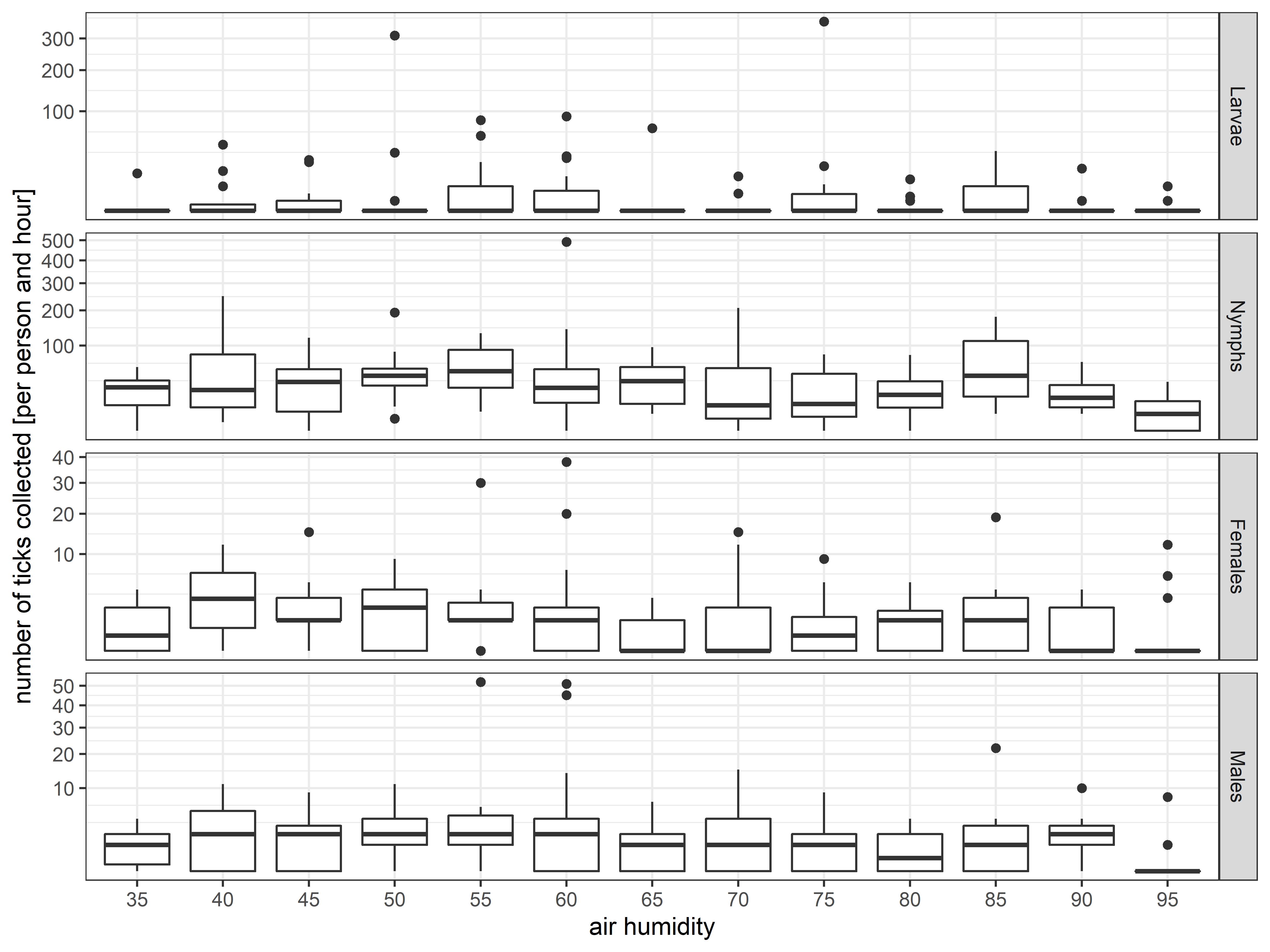

Supplement: Supplementary file 1 — Numbers of ticks caught per humidity and age group [per person and hour] (JPEG 884 kb) [file 436_2020_6666_MOESM1_ESM.jpeg]
