# Supplementary material for: Research paper on abiotic factors and their influence on Ixodes ricinus activity—observations over a two-year period at several tick collection sites in Germany
Source: Parasitol Res. 2020 Mar 26;119(5):1455–66. doi: 10.1007/s00436-020-06666-8 (PMC7184057; doi:10.1007/s00436-020-06666-8)
Supplement: Supplementary file 2 — Sites for measuring tick activity (DOCX 13 kb) [file 436_2020_6666_MOESM2_ESM.docx]

**Table S1: Sites for checking tick activity**

| **Federal state** | **place** | **habitat** | **GPS data** |
| --- | --- | --- | --- |
| Thuringia | Jena/ Kunitz | forest/meadow | N 50° 57' 47. 8" / E 11° 38' 44. 7" |
| Thuringia | Jena/ Steinkreuz 1 | forest | N 50° 54' 50. 3'' / E 11° 38' 23. 0'' |
| Thuringia | Jena/ Steinkreuz 2 | forest | N 50° 54' 47. 6" / E 11° 38' 31. 2" |
| Thuringia | Jena/Jahnwiese | meadow | N 50° 55' 34. 3'' / E 11° 33' 01. 4" |
| Thuringia | Jena/Orchideenwiese | meadow | N 50° 55' 08. 3" / E 11° 31' 48. 0" |
| Mecklenburg-Western Pomerania | Groß Quassow | forest | N 53° 18' 39. 0'' / E 13° 00' 05. 6'' |
| Mecklenburg-Western Pomerania | Vosswinkel | meadow | N 53° 18' 53. 6'' / E 13° 02' 14. 4'' |
| Saarland | Rohrbach/ St. Ingbert/ Glashütter Weiher | forest | N 49° 17' 52. 4" / E 07° 10' 47. 2" |
| Saarland | Spiesen/ Spiesermühle | meadow | N 49° 17' 46. 6" / E 07° 09' 33. 3" |
| North Rhine- Westphalia | Büren/ Paderborn | forest | N 51° 33' 28. 4" / E 08° 31' 20. 9" |
| North Rhine- Westphalia | Oestereiden/Soest | meadow | N 51° 33' 18. 9" / E 08° 27' 07. 3" |
| Lower Saxony | Rotenburg/Rotschule | forest | N 53° 05' 36. 6" / E 09° 22' 26. 9" |
| Lower Saxony | Rotenburg/Unterstedt | forest | N 53° 04' 19. 2" / E 09° 21' 06. 9" |
| Lower Saxony | Holtum-Geest | forest | N 53° 00' 01. 9" / E 09° 19' 35. 0" |
| Lower Saxony | Rotenburg/ Weichelsee | meadow | N 53° 07' 06. 9" / E 09° 22' 27. 8" |
| Bavaria | Loderhof/Tittling | forest | N 48° 44' 21. 2" / E 13° 20' 43. 3" |
| Bavaria | Loderhof/Tittling | meadow | N 48° 44' 16. 6" / E 13° 20' 48. 0" |
| Baden-Wuerttemberg | Hausach | forest | N 48° 16' 04. 1" / E 08° 10' 03. 0" |
| Baden-Wuerttemberg | Hausach | meadow | N 48° 16' 02. 7" / E 08° 10' 06. 7" |
