# Supplementary material for: Research paper on abiotic factors and their influence on Ixodes ricinus activity—observations over a two-year period at several tick collection sites in Germany
Source: Parasitol Res. 2020 Mar 26;119(5):1455–66. doi: 10.1007/s00436-020-06666-8 (PMC7184057; doi:10.1007/s00436-020-06666-8)
Supplement: Supplementary file 3 — Ticks collected in 2009 and 2010 at all sites (DOCX 14 kb) [file 436_2020_6666_MOESM3_ESM.docx]

**Table S2: Collected ticks in 2009 and 2010 at all sites**

| **year** | **Federal state** | **n** | **female** | **male** | **nymphs** | **larvae** | **total** | **Larvae (estimated)** |
| --- | --- | --- | --- | --- | --- | --- | --- | --- |
| **2009** | MW | 6 | 88 | 103 | 1.716 | 345 | 2.252 | 0 |
|  | LS | 6 | 83 | 97 | 913 | 37 | 1.130 | 0 |
|  | NRW | 4 | 4 | 5 | 454 | 295 | 758 | 790 |
|  | SL | 6 | 114 | 91 | 2.719 | 406 | 3.330 | 220 |
|  | TH | 16 | 142 | 132 | 256 | 341 | 871 | 60 |
|  | total 2009 | **38** | **431** | **428** | **6.058** | **1.424** | **8.341** | **1.070** |
| **2010** | BW | 5 | 158 | 192 | 2.261 | 101 | 2.712 | 800 |
|  | BY | 4 | 89 | 71 | 820 | 93 | 1.073 | 0 |
|  | MW | 6 | 46 | 77 | 1.860 | 156 | 2.139 | 0 |
|  | LS | 7 | 61 | 82 | 1.620 | 172 | 1.935 | 0 |
|  | NRW | 2 | 6 | 14 | 209 | 258 | 487 | 0 |
|  | SL | 4 | 41 | 25 | 608 | 48 | 722 | 0 |
|  | TH | 9 | 29 | 37 | 144 | 10 | 220 | 0 |
|  | total 2010 | **37** | **430** | **498** | **7.522** | **838** | **9.288** | **800** |
| **total 2009/2010** |  | **75** | **861** | **926** | **13.580** | **2.262** | **17.629** | **1.870** |

BW: Baden-Württemberg; BY: Bavaria; MW: Mecklenburg-Western Pomerania; LS: Lower Saxony; NRW: North-Rhine Westphalia; SL: Saarland; TH: Thuringia
